# Unveiling Peripherally Inserted Central Catheter Fractures and Related Complications in the Neonatal Intensive Care Unit: A Concise Review

**DOI:** 10.7759/cureus.47572

**Published:** 2023-10-24

**Authors:** Palanikumar Balasundaram, Michelle H Lucena, Lan Jiang, Suhas Nafday

**Affiliations:** 1 Department of Pediatrics, Division of Neonatology, Javon Bea Hospital-Riverside, Mercy Health system, Rockford, USA; 2 Department of Pediatrics, Division of Neonatology, Baylor College of Medicine, Texas Children’s Hospital, Houston, USA; 3 Department of Pediatrics, Children’s Hospital at Montefiore, Albert Einstein College of Medicine, Bronx, USA; 4 Department of Pediatrics, Division of Neonatology, Jack D. Weiler Hospital, Children’s Hospital at Montefiore, Albert Einstein College of Medicine, Bronx, USA

**Keywords:** neonatal intensive care unit (nicu), catheter jamming, fracture, picc line complication, peripherally inserted central catheter (picc)

## Abstract

Peripherally inserted central catheters (PICCs) have become popular over tunneled catheters in neonatal intensive care units (NICUs) due to their ease of use and convenience. Although rare, a PICC fracture can be a severe and potentially fatal complication. This narrative review aims to identify factors predisposing neonates to PICC fracture and related complications, such as catheter jamming, and explore strategies for preventing and detecting this complication. A thorough search of PubMed and Google Scholar was conducted using relevant keywords to identify articles discussing PICC fracture in neonates. The review encompassed English-language literature on PICC fracture in neonates, with additional pertinent publications identified through citation searching.

The incidence of PICC fracture in neonates varies from less than 1% to 10%, with a higher risk associated with prolonged catheterization, lower gestational age and lower birth weight, and the use of multi-lumen catheters. PICC fractures can occur during insertion, maintenance, or removal. Factors such as catheter duration, gestational age, birth weight, and catheter type increase the risk of PICC fracture. Excessive syringe pressure, securement failure, and excessive force during removal are contributing factors. Catheter fatigue and thin-walled catheter design are common causes of breakage. Preventive measures include proper training of healthcare providers, regular monitoring, early recognition, and prompt catheter removal upon fracture. Preventing and detecting PICC fractures is crucial for neonatal safety. Vigilance during insertion, maintenance, and removal, along with care to avoid excessive force during removal and high pressure during flushing, can help prevent catheter breakage. More research is required to improve prevention strategies for PICC fractures in neonates.

## Introduction and background

Peripherally inserted central catheters (PICCs) have replaced tunneled catheters since the 1970s. The utilization of PICCs in neonatal intensive care units (NICUs) has witnessed a remarkable surge, attributed to their reduced complication rates and less invasive insertion procedures in contrast to surgically placed non-tunneled and tunneled catheters. The availability of smaller PICCs (1 Fr and 2 Fr) has grown over the last two decades. The most common complications are catheter-related bloodstream infections, occlusion, thrombosis, infiltration, unintentional removal, pleural effusion, pericardial effusion, catheter fracture, and embolization [1-6]. The younger the gestational age and lower the birth weight, the higher the rates of PICC complications observed. A PICC fracture is the term used to describe the occurrence of a break or separation in the catheter tubing that has been inserted into an infant’s vein. PICC fracture is uncommon but life-threatening. This narrative review aims to identify and characterize potential predisposing factors for PICC fracture and related complications, such as catheter jamming in the neonatal population, and evaluate methods for preventing these issues.

## Review

Search methodology

**Figure 1:**
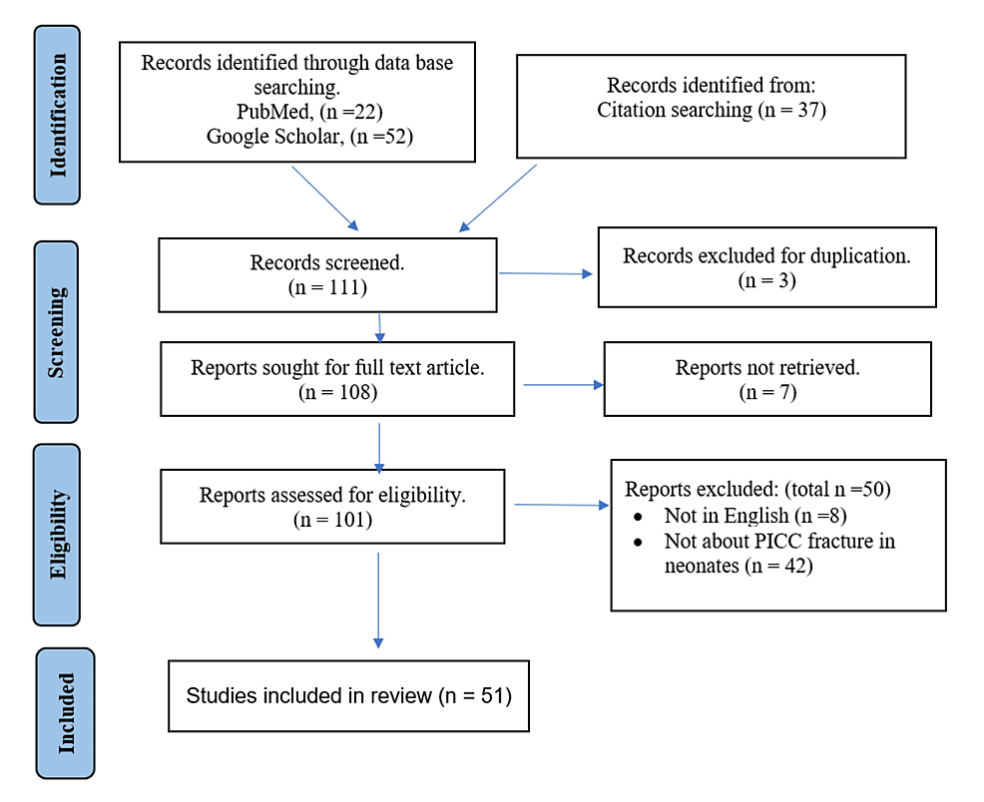
Flow diagram of the study selection process.

A comprehensive search was done through PubMed and Google Scholar to identify publications about PICC fracture and catheter jamming in the neonatal population until April 2023. The keywords “PICC,” “peripherally inserted central catheter,” “central catheter,” “fracture,” “embolization,” “catheter jamming,” “difficult removal,” “neonate,” and “NICU” were used. Citation searching was also employed to evaluate more publications. Articles were included for the review if they discussed PICC fracture or catheter jamming in the NICU. Publications in languages other than English were excluded. Ethical clearance was unnecessary for this narrative review, as it involved consolidating and examining existing data from previous studies without direct engagement with human subjects or collecting new primary data. Figure 1 summarizes the search methods and the study selection process.

PICC usage in the NICU

A PICC is a flexible and slender catheter made of biocompatible materials, such as silicone or polyurethane, inserted into the body through the skin. PICCs have been extensively utilized in NICUs to provide medical and nutritional assistance for infants who are critically ill or have extreme and very low birth weight. PICCs are available in sizes ranging from 1 Fr to 3 Fr in single or double-lumen configurations for use in the NICU. Smaller vessel and catheter diameters imply increased technical difficulties during insertion, maintenance, and removal for the neonatal population compared to larger children and adults.

PICC complications in neonates

Despite the benefits of using a PICC instead of a traditional central vein catheter (CVC), the insertion and maintenance of PICCs can be challenging. Catheter fractures are more prevalent in PICCs than in traditional CVCs. PICCs are associated with multiple other complications, such as migration or dislodgement, air embolism, bleeding, extravasation, arrhythmia, cardiac tamponade, venous or right atrial perforation, pleural or pericardial effusion, hydrothorax, phrenic nerve injury or diaphragmatic paralysis, inadvertent arterial cannulation, venous thrombosis, mechanical or chemical phlebitis, and resistance to removal, also known as jamming [1-6].

PICC fracture

The incidence of PICC fractures is approximately 6.7 per 1,000 PICCs in pediatrics [4], and in neonates, it varies widely across studies, ranging from less than 1% to as high as 10% [2,7-11]. Table 1 summarizes the published data on the frequency of PICC fractures in the NICU.

**Table 1:** Published data on the PICC fracture incidence in the NICU. PICC = peripherally inserted central catheter; NICU = neonatal intensive care unit

Study	Observation period	Sample size	Fracture rate	Fracture location
Pet et al. 2019 [2]	42 months	1,234	5% (62/1,234)	23 in the upper extremity and 39 in the lower extremity (odds ratio of 0.79 (0.47–1.34) was not statistically significant)
Costa et al. 2013 [10]	12 months	237	6.7% (16/237)	Centrally placed PICC (15/207) had more fractures than non-central catheters (1/30)
Tsai et al. 2011 [11]	4 years	808	3.2 % (26/808)	Catheters with a stiffening stylet and 22 G introducer (2.5%–13/518) had a higher fracture rate than single-lumen catheters with 24 G introducer (4.5%–13/290)
Lloreda-García et al. 2016 [12]	6 years	193	2% (4/193)	Not reported
Wrightson et al. 2013 [1]	5 years	626	0.5% (3/626)	1 upper extremity and 2 lower extremity
Corzine et al. 2010 [7]	5 years	491	1.7% for central catheters (8/462) and 3.4% for non-central catheters (1/29)	1.6 per 1,000 catheter days for the arm, 1.9 per 1,000 catheter days for the leg
Paulson et al. 2008 [8]	7 years	159	1.9% (3/159)	Not reported
Evans et al. 1999 [9]	51 months	140	10% (14/140)	7 cracked hubs and 7 tears at the hub-catheter junction

According to Pet et al., there was no statistically significant difference in the risk of PICC fracture between upper and lower extremity catheters [2]. Catheter strength depends on size and material. Polyurethane has higher tensile strength than silicone, but both can fracture with improper care. Gomes de Souza et al. reported that the probability of PICC fracture is higher in silicone catheters than in polyurethane [13]. Factors associated with increased risk of PICC fracture include longer duration of catheterization, lower gestational age and birth weight, and stiff or multi-lumen catheters. Tsai et al. have documented that PICC insertion that takes longer than 60 minutes and PICC placement for more than 30 days are correlated with greater rates of catheter-related complications, including fractures of the PICC [11].

PICC fractures can occur during insertion, maintenance, or removal. During insertion, the catheter may fracture if it is withdrawn while the insertion needle is still in place or if the patient moves significantly [14-16]. The risk of PICC fracture increases when excessive syringe pressure is applied, especially when using small-volume syringes for infusion or flushing against resistance [17,18]. Another factor during maintenance associated with PICC fractures in the outer segment is the failure to secure the line or excessive force during dressing changes [19-21]. Sharp objects such as clamps and adhesive tape on the catheter can cause PICC fractures. During removal or adjusting the depth, PICC fracture is possible if excessive force is applied [22-24].

Signs of a fractured catheter in neonates may include local site swelling, tenderness, redness, leakage, and resistance during insertion and removal [25]. PICC fracture can be an incidental finding without symptoms and may be detected on routine imaging or removal [26-29].

Table 2 summarizes cases of PICC fractures in NICU settings from various journal reports. Notable findings include PICC fractures occurring within a few days to months after insertion, with the risk of embolizing catheter fragments into ventricles and critical arteries such as pulmonary arteries. Additionally, lower gestational age and birth weight seem to be associated with an elevated likelihood of PICC fractures. Preceding events leading to PICC fractures exhibited diversity, encompassing symptoms such as catheter swelling and blood stains at insertion sites, and, in certain instances, catheter embolization into the pulmonary artery. The retrieval methods range from cardiac catheterization and endovascular techniques to more invasive procedures such as thoracotomies. These cases emphasize the critical importance of monitoring and adopting suitable retrieval strategies for PICC fractures among neonatal patients.

**Table 2:** PICC fracture cases reported in various journals within the NICU setting. PICC = peripherally inserted central catheter; NICU = neonatal intensive care unit

Author/year	Patient characteristics		Catheter details			Events preceding the PICC fracture	Fracture day	Site of embolization	Retrieval method
	Gestation at birth	Birth weight	Size	Site	Insertion day				
Bishnoi et al. 2023 [30]	32 weeks	1,100 g	-	-	-	Transferred for the retrieval of the embolized fragment	4	Left pulmonary artery	Cardiac catheterization
Minghui et al. 2015 [31]	32 weeks	1,460 g	1.9 Fr	Median cubital	4 days	Bloodstain at the site of insertion	2.5 months	Right pulmonary artery	Cardiac catheterization. Developed cardiac tamponade
Kalra et al. 2012 [32]	31 weeks	1,090 g	1.9 Fr	Left antecubital	1 day	Swelling in the axilla	12 days	Right ventricle	Cardiac catheterization
Robbins et al. 2008 [33]	32 weeks	1,450 g	1.9 Fr	Right basilic	5 days	Loop and knot noted on X-ray. Snapped on removal	12 days	Basilic and axillary vein	Percutaneous endovascular retrieval
Chiang et al. 2006 [18]	28 weeks	1,120 g	-	Right antecubital	4 days	A fractured fragment noted on X-ray	5 days	Right ventricle	Cardiac catheterization
Pigna et al. 2004 [15]	36 weeks	1,700 g	27 G	Basilic vein	4 days	A fracture fragment noted on a routine X-ray	7 days	Left pulmonary artery (peripheral branch)	Not removed due to the risks associated with the procedure
Young et al. 2003 [16]	28 weeks	800 g	1.9 Fr	Right antecubital	10 days	Unsuccessful initial placement on the right and later placed on the left arm	10 days	Right atrium	Cardiac catheterization
Andrews et al. 2002 [24]	Preterm	-	-	Right antecubital	-	The catheter snapped on removal	6 weeks	Pulmonary artery	Cardiac catheterization with snare
Bagna et al. 2001 [29]	Case 1: 30 weeks. Case 2: 27 weeks	Case 1: 1,560 g. Case 2: 1,140 g	-	Left basilic	Case 1: 6 days. Case 2: 4 days	A routine echocardiogram showed a fracture	Case 1: 9 days. Case 2: 6 days	Right ventricle	Case 1: Femoral vein cannulation. Case 2: Thoracotomy
Hsu et al. 1998 [26]	26 weeks	780 g	23 G	Left femoral vein	4 days	The catheter was removed due to occlusion and found to be shorter than expected	28 days	Left common iliac vein	Endovascularly with a snare loop
Abbruzzese et al. 1998 [28]	27 weeks	1,060 g	0.6 mm	Left antecubital	4 days	An echocardiogram confirmed that a distal fragment had embolized	5 days	Distal right pulmonary artery	Intrapericardial removal with a nerve hook using a median sternotomy approach
Ochikubo et al. 1996 [14]	28 weeks	-	-	-	-	Fracture of the catheter during insertion	-	Right atrium	Cardiac catheterization
Massin et al. 1997 [23]	30 weeks	1,395 g		Right basilic antecubital	2 days	PICC adjustment for deep insertion, and X-ray confirmed distal piece embolization	4 days	Left atrium	cardiac catheterization with a helical basket
Hwang et al. 1997 [19]	Case 1: 31 weeks. Case 2: 31 weeks	Case 1: 1,750 g. Case 2: 1,360 g	2 Fr	Right arm	Not mentioned	PICC breakage during routine care	Case 1: 28 days. Case 2: 53 days	Right pulmonary artery	Modified snare guidewire through the right femoral vein
Bautista et al. 1995 [34]	31 weeks	1,630 g	2 Fr	Right antecubital	5 days	The catheter snapped on attempted removal due to occlusion	53 days	The catheter was tethered at the cephalic vein, with no embolism	Surgical exploration of the right cephalic vein
Watkin et al. 1994 [25]	35 weeks	-	-	Right long saphenous	4 days	The catheter was removed due to leaking and found snapped	12 days	Bifurcation of the pulmonary artery	Cardiac catheterization was unsuccessful, and sternotomy was required
Khilnani et al. 1990 [27]	37 weeks	2.4 kg	1.9 Fr	Right external jugular vein	17	The catheter was noted embolized on a routine X-ray	22 days	Left pulmonary artery	After the catheter snare failed, it was removed with thoracotomy and pulmonary arteriotomy
Gladman et al. 1990 [35]	Case 1: 29 weeks. Case 2: 26 weeks	Case 1: 1,705 g. Case 2: 700 g	-	Case 1: Scalp. Case 2: Antecubital	Case 1: 17 days. Case 2: 24 days	Case 1: Sepsis (snapped on removal). Case 2: Swollen limb (snapped on removal)	Case 1: 33 days. Case 2: 29 days	Proximal segment tethered at the insertion site, no embolism	Surgical exploration with a small incision at the tethered site

Risk factors associated with PICC fracture

**Figure 2:**
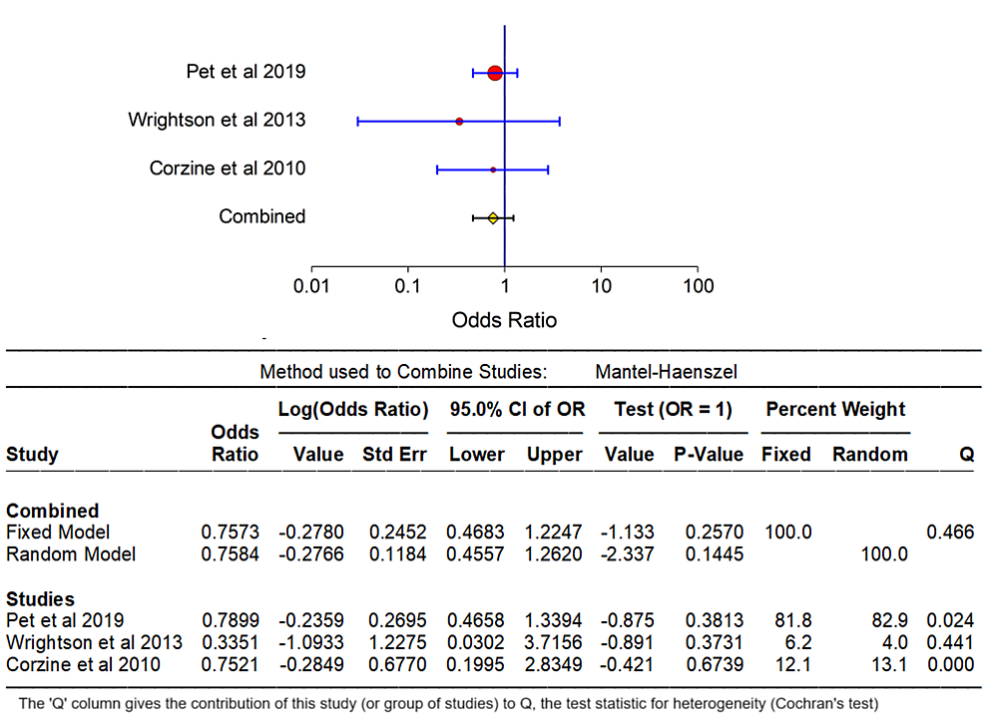
Upper vs. lower extremity PICC fracture risk. PICC = peripherally inserted central catheter

**Figure 3:**
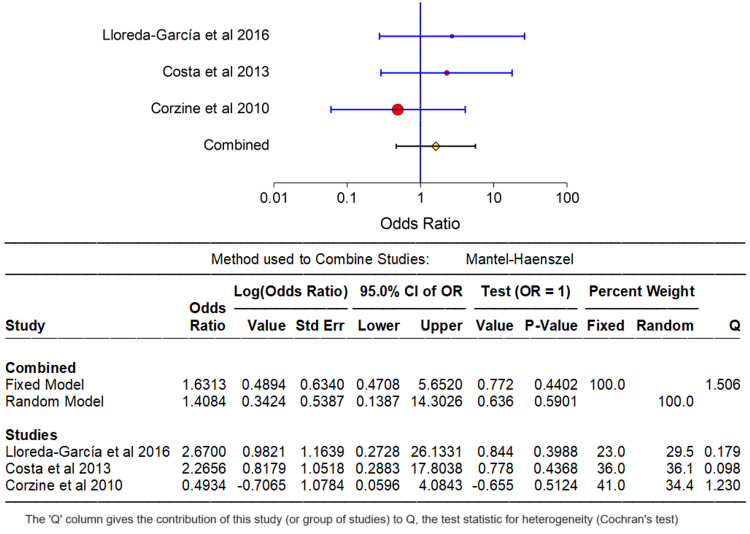
Central vs. non-central PICC fracture risk. PICC = peripherally inserted central catheter

A comprehensive analysis was conducted on the available data, stratifying the results into upper vs. lower extremity and central vs. non-central catheter insertion sites concerning PICC fracture. Comprehensive summary statistics and forest plots were generated for these variables using the NCSS 2023 Statistical Software (NCSS, LLC, Kaysville, Utah, USA), contributing to a robust understanding of outcomes associated with these distinctions. Figure 2 and Figure 3 forest plot images visually depict the outcomes, reinforcing the conclusion that there is no statistically significant difference in PICC fracture rates among central vs. non-central and upper vs. lower extremity catheter insertions. It is noteworthy that variables such as gestational age, birth weight, catheter size, duration of catheterization, and catheter type posed challenges in the analysis due to limited literature on PICC fracture. Nonetheless, valuable insights into PICC fracture were extracted through a qualitative analysis of the existing case reports and case series, though a formal statistical synthesis was not performed.

There is a strong association between catheter fracture, the length of catheter placement, and line complications, such as blockage or leakage [4]. The primary cause of catheter breakage is generally attributed to catheter fatigue. Thin-walled PICCs designed for premature infants are more prone to breakage than wider bore catheters. The use of antiseptic solutions before catheter insertion may weaken the catheter material. The catheter is susceptible to fracture at multiple points, including the catheter hub, internal catheter, and extension tubing. Fractures in PICCs typically occur at the junction between the thin polyurethane section and the wider microbore tubing. The external tubing and hubs are subject to wear and tear from repeated use. The catheter-hub junction is prone to fracture if excessive and sudden tension is applied while weighing the neonate or during skin-to-skin care [7]. Mechanical manipulation, particularly rotational torque or twisting, is more likely to induce fatigue and lead to breakage than linear bending.

Additionally, flushing blocked lines under high pressure, using too much manual force, or small syringes (<5 mL) may contribute to catheter breakage. The risk of mechanical complications, including PICC fracture, is similar in the upper and lower extremities [36]. However, Chang et al. reported a higher risk of catheter fracture in pediatric patients with catheters placed distally on the forearm [3]. This finding was not confirmed in another study that demonstrated the same fracture tension of proximal and distal PICCs [37]. Although most pediatric studies have not found any difference in post-insertion complications between silicone and polyurethane PICCs, Gomes de Souza et al. reported a higher occurrence of non-elective removals, extravasations, and fractures among neonates with silicone catheters in contrast to polyurethane ones [13]. Using larger PICCs can elevate the likelihood of venous occlusion and thrombosis, while smaller catheters may cause more mechanical issues, including luminal occlusion and fractures [38]. It is recommended to prioritize single-lumen catheters as they may have a lower likelihood of causing complications than multi-lumen catheters [39].

Difficult PICC removal in the NICU

Among the mechanical complications of PICC, catheter jamming is a concern. The term “catheter jamming” refers to a situation where a catheter is pulled with considerable force close to its fracture tension but remains stuck and cannot be removed. Several reasons are associated with the difficulty of removing a catheter, such as venous spasms [40], venous thrombus with or without infection trapping the catheter within the vein wall [34], or phlebitis [35,40]. Stimulation of the vessel wall during catheter removal can result in severe vasospasm, which can be felt as a cord. A fibrin sheath can occasionally explain a difficult removal after partial extraction [41]. Coiling or knotting is also responsible for excessive resistance during catheter removal [33,42]. Despite these challenges, most PICCs can be removed over time, as long as aggressive traction is avoided to prevent catheter fracture and venous damage. Table 3 provides an overview of cases where PICC removal posed challenges in the NICU.

**Table 3:** Summary of published articles on catheter jamming in the NICU (10 cases). PICC = peripherally inserted central catheter; NICU = neonatal intensive care unit

Author/Year	Gestational age at birth	Birth weight	PICC size	Site	Insertion day	Case scenario	PICC removal day	Retrieval method
Chen et al. 2020 [36]	27 weeks	1,070 g	1.9 Fr	Right saphenous	19 days	Erythema and swelling at the insertion site. Difficult while removal	23 days	Warm compress + topical phentolamine
Zhou et al. 2016 [42]	29 weeks	1,222 g	2 Fr 24 G	Left long saphenous	113 days	Difficulty in removal due to knot formation	141 days	Transjugular under general anesthesia
Robbins et al. 2008 [33]	32 weeks	1,450 g	1.9 Fr	Right basilic	5 days	Loop and knot noted on the X-ray. Snapped on attempted removal	12 days	Percutaneous endovascular retrieval
Gladman et al. 1990 (4 cases) [35]	Case 1: 29 weeks. Case 2: 26 weeks. Case 3: 29 weeks. Case 4: 26 weeks	Case 1: 1,705 g. Case 2: 700 g. Case 3: 1,060 g. Case 4: 800 g	-	Case 1: Scalp. Case 2: Antecubital. Case 3: Right dorsum	Case 1: 17 days. Case 2: 24 days. Case 3: 25 days. Case 4: 30 days	Cases 1 and 4: Sepsis. Case 2: Swollen limb. Case 3: Erythema at the insertion site. All 4 cases: Catheter jamming was noted, causing snapping in cases 1 and 2	Case 1: 28 days. Case 2: 29 days. Case 3: 25 days. Case 4: 44 days	Cases 1 and 2: Surgical exploration. Cases 3 and 4: Traction with splint method. All catheter tips grew *S. epidermidis*
Bautista et al. 1995 (3 cases) [34]	Case 1: 31 weeks. Case 2: 27 weeks. Case 3: 27 weeks	Case 1: 1,630 g. Case 2: 1,000 g. Case 3: 1,045g	2 Fr	Case 1: Right antecubital vein. Case 2: Left posterior auricular vein. Case 3: Right antecubital	Case 1: 5 days. Case 2: 19 days. Case 3: 3 days	All cases had catheter firmly attached, possibly due to phlebitis, and in case 1, the catheter snapped	Case 1: 53 days. Case 2: 70 days. Case 3: 37 days	Surgical exploration of the right cephalic vein (case 1), a small incision at the insertion site (cases 2 and 3) The catheter tip grew *S. epidermis* in case 3

Preventive measures for PICC fracture in the NICU

Specific measures can be taken during insertion, maintenance, and removal stages to prevent PICC fractures in the NICU.

Insertion Stage

**Figure 4:**
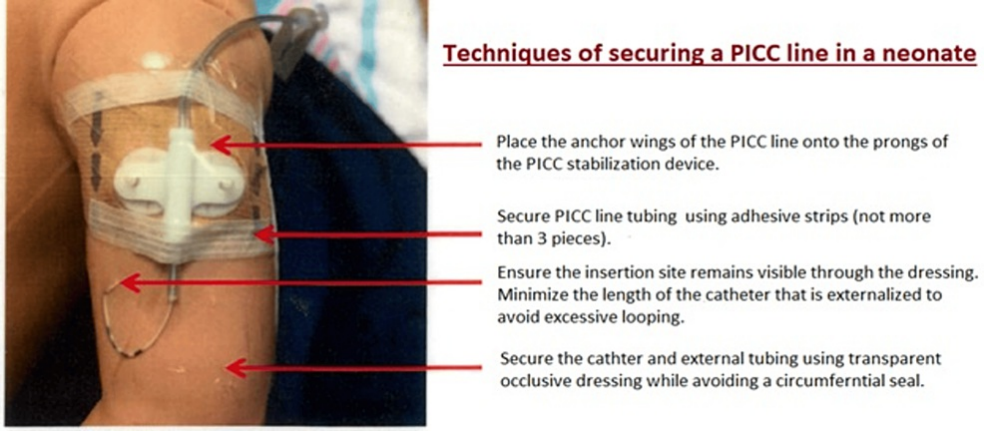
Techniques of securing a PICC line in neonates. Image credit: Palanikumar Balasundaram, Michelle H. Lucena, Lan Jiang, and Suhas Nafday. PICC = peripherally inserted central catheter

**Figure 5:**
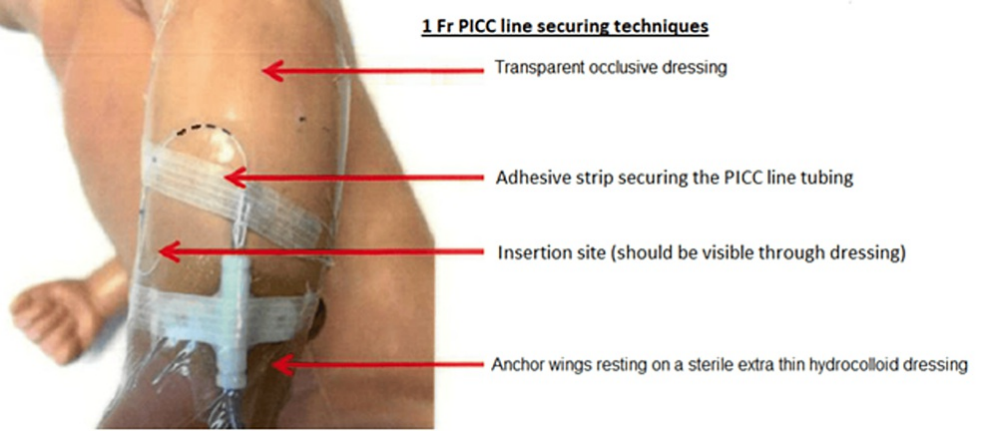
1 Fr PICC line securing techniques. Image credit: Palanikumar Balasundaram, Michelle H. Lucena, Lan Jiang, and Suhas Nafday. PICC = peripherally inserted central catheter

Before PICC insertion, a thorough evaluation of the patient’s need for analgesia or sedation to reduce agitation and promote a smoother procedure is essential. Furthermore, it is imperative to assess the catheter’s length, insertion site, and the condition of the vein. Before inserting a PICC line, a comprehensive examination of the catheter for any signs of leaks or breakage should also be conducted. Retracting the catheter through the introducer needle should be avoided to prevent catheter shearing. One way to reduce tension on the catheter is to secure it under the dressing and use a T-connector. Additionally, fixing the catheter in a standardized manner, with the external part of the catheter in an “S” shape or a loop, can also be effective [31]. To prevent the catheter from moving and developing kinks that may cause breakage, the catheter and the hub should be firmly secured using skin closure strips and a transparent occlusive dressing [7-9]. Corzine et al. found that securing the extension tubing with a second layer of a sterile semi-permeable dressing reduced PICC fracture in a NICU from 3.5% to 1%. The technique for securing the PICC is illustrated in Figure 4 and Figure 5.

Maintenance Stage

High-pressure injections through PICC can damage the catheter wall and cause line fracture [4]. Infusion pumps should be used strictly per manufacturer instructions to avoid excessive pressure. Providers and nurses should avoid forcefully infusing fluids using syringes, and if resistance is encountered, flushing should be avoided. If flushing is required, using a syringe with a capacity greater than 5 mL is recommended [43]. Catheters should be removed as soon as they are no longer needed, as prolonged use of PICCs increases the risk of breakage. However, the routine removal and reinsertion of PICCs may pose risks and incur costs. Monitoring the PICC tip position regularly through radiographs or ultrasound to ensure the optimal placement is crucial. Handling them as little as possible is recommended to minimize the fracture risk to the PICC and external tubing. In addition, a critical aspect of the maintenance phase involves frequent education of nurses and providers on proper PICC line usage and maintenance. According to the findings of Garduno-Crespo et al., the risk of breakage is higher when PICC care, including dressing change, is performed every seven days compared to a 10-day interval [44]. If the fluid administered is total parenteral nutrition, lipids, or dextrose >10%, the intravenous (IV) tubing should be replaced every 24 hours; otherwise, every 96 hours is sufficient [8]. To ensure the catheter remains patent, heparin should be added to the fluids at a ratio of 0.5 to 1 U/1 mL and administered continuously at a minimum rate of 1 mL/hour for preterm neonates and 2 mL/hour for term neonates [45]. Extension tubing with a clamp is recommended to avoid clamping the actual catheter. PICC with a diameter of less than 2 Fr should not be used for blood administration or sampling. Additionally, tourniquets, IV boards, and blood pressure cuffs should be avoided on the extremities with a catheter. Enhancing catheter securing techniques and regularly employing X-rays to track the catheter’s tip position helps prevent severe complications, including PICC fractures.

Removal Stage

Only trained personnel should remove PICCs, and care should be taken when removing the occlusive dressing. To ensure the safe removal of the PICC, it is recommended to hold the catheter at the insertion point instead of the hub and avoid applying significant force. Excessive force can cause the catheter to fracture, whereas too little force can result in the pseudo-stalling of the catheter. Removing the line with slow and gentle continuous traction is advisable [37]. The catheter is slowly drawn in 2-3 mm increments until it reaches its tip, followed by a thorough inspection of the entire catheter. If significant resistance is encountered while attempting PICC removal, the provider should seek expert advice, as the PICC may break.

Approach to difficult PICC removal

When faced with challenges in removing a PICC, consider using imaging techniques such as X-rays and ultrasounds to identify knots, kinks, or thrombi. There are several methods available to address difficult PICC removal.

1. One approach involves gently pulling the catheter and securing it to the insertion site, repeating this process every few hours. If the catheter cannot be removed within four hours, it may be required to obtain an X-ray to determine its location. However, catheter snapping is a risk when this technique is applied [22,34,35].

2. Another method is to apply warm compresses along the vein’s tract for 30 minutes, followed by an attempt to remove the catheter. Applying a warm compress with warm saline can cause blood vessels to dilate and enhance blood circulation. This process can be repeated every 8-12 hours. This approach has less potential for catheter breakage [40,46,47]. Solutions safe for peripheral veins, such as normal saline or dextrose (<12.5%) with heparin, should be used while waiting for removal.

3. Combining warm and moist compresses with topical phentolamine can aid in the removal of a stuck catheter in neonates [48]. Phentolamine, a vasodilator, relieves vasospasm by reducing peripheral vascular resistance.

4. Additional techniques include flushing and rotating the catheter, repositioning the extremity before the removal attempt, and massaging the skin overlying the vein [40,49].

When the catheter cannot be easily removed, gentle and gradual traction, warm compresses, rotating the catheter, and allowing time to pass are recommended.

Approach to snapped PICC during removal

In the event of a snapped PICC during removal, the outer segment of the catheter should be grasped to prevent embolism. In the event of an embolism, it is crucial to take measures to prevent the catheter fragment from moving deeper into the central circulation. This can be achieved by applying digital pressure or a tourniquet to the affected extremity [50]. However, it is crucial to ensure that the tourniquet is not excessively tight, which may obstruct arterial blood flow. The infant should be placed on the right side to trap the catheter within the right heart. The patient should be kept still and radiographic confirmation of the catheter fragment’s location should be requested. Prompt removal of a fractured catheter fragment is critical to minimize the risk of complications such as arrhythmia, blood clot formation, and catheter embedding in the vessel walls [51]. To remove catheters in the peripheral circulation, venotomy should be performed. However, if catheters have already embolized to the central circulation, their removal may require interventional radiology, interventional cardiology, or surgical procedures [30-32].

## Conclusions

PICC fractures are an uncommon but life-threatening complication in neonates, and their prevention and early detection are crucial to ensure neonatal safety. More studies are needed to confirm the association between PICC fracture risk and factors such as extended catheterization periods, lower gestational age, lower birth weight, and the utilization of rigid or multi-lumen catheters. To prevent PICC fractures, healthcare providers should be vigilant during the insertion, maintenance, and removal of these lines and avoid excessive force and high-pressure flushing. Early detection and management of PICC fractures are essential to minimize morbidity and mortality. Further research is needed to understand the risk factors better and develop more effective prevention strategies for PICC fractures in neonates.
